# Impact of various aligner auxiliaries on orthodontic activity: A systematic review and network *meta*-analysis

**DOI:** 10.1016/j.sdentj.2023.10.013

**Published:** 2023-10-27

**Authors:** Mohammad Khursheed Alam, Mohammad Younis Hajeer, Abedalrahman Shqaidef, Haytham Jamil Alswairki, Ahmed Ali Alfawzan, Deepti Shrivastava, Kumar Chandan Srivastava, Marco Cicciù, Giuseppe Minervini

**Affiliations:** a Orthodontic Division, Preventive Dentistry Department, College of Dentistry, Jouf University, Sakaka 72345, Saudi Arabia; b Department of Dental Research Cell, Saveetha Dental College and Hospitals, Saveetha Institute of Medical and Technical Sciences, Chennai, India; c Department of Public Health, Faculty of Allied Health Sciences, Daffodil International University, Dhaka, Bangladesh; d Department of Orthodontics, University of Damascus Faculty of Dentistry, Damascus, Syria; e Department of Clinical Sciences, Center of Medical and Bio-Allied Health Sciences Research, College of Dentistry, Ajman University, Ajman, United Arab Emirates; f School of Dental Sciences, Universiti Sains Malaysia, Kota Bharu 16150, Malaysia; g Department of Preventive Dentistry, College of Dentistry in Ar Rass, Qassim University, Ar Rass 52571, Saudi Arabia; h Periodontics Division, Preventive Dentistry Department, College of Dentistry, Jouf University, Sakaka 72345, Saudi Arabia; i Department of Oral & Maxillofacial Surgery & Diagnostic Sciences, College of Dentistry, Jouf University, Sakaka 72345, Saudi Arabia; j Department of Oral Medicine and Radiology, Saveetha Dental College, Saveetha Institute of Medical and Technical Sciences, Saveetha University, Chennai 602105, India; k Department of Biomedical and Surgical and Biomedical Sciences, Catania University, 95123 Catania, Italy; l Saveetha Dental College & Hospitals, Saveetha Institute of Medical & Technical Sciences, Saveetha University, Chennai 600 077, India; m Multidisciplinary Department of Medical-Surgical and Dental Specialties, University of Campania, Luigi Vanvitelli, 80138 Naples, Italy

**Keywords:** Orthodontic treatment, Aligner auxiliaries, Elastic ligatures, Tooth movement

## Abstract

**Background:**

It is imperative to analyze the forces and moments produced by various auxiliaries in order to select the optimal attachments and, eventually, to maximize the efficacy and efficiency of orthodontic therapy. Through this investigation, we aimed to highlight the impact of various aligner auxiliaries on orthodontic activity in patients undergoing orthodontic treatment on a pre/post treatment protocol basis.

**Methods:**

After a thorough search of the online journals, a total of 482 documents were found using keywords such as “Orthodontic Treatment”, “Aligner Auxiliaries”, “Elastic Ligatures” and “Tooth Movement.” The database research, elimination of duplicate studies, data extraction and risk of bias were performed by the authors independently. This systematic review and network *meta*-analysis included prospective studies and clinical trials to evaluate research that had looked at the impact of various aligner auxiliaries on orthodontic activity in patients undergoing orthodontic treatment.

**Results:**

Eight investigations of varying designs were selected for this review. The majority of investigations revealed that aligner auxiliaries significantly improve anterior root torque, rotation, and mesio-distal (M−D) movement, as well as posterior anchoring. They also significantly improved anterior root rotation. However, few studies have presented inconsistent or non-statistically significant findings.

**Conclusion:**

Auxiliaries for aligners also appear to improve extrusion and other orthodontic movements, but there is insufficient evidence to support these claims. No research has examined posterior bucco-lingual expansion or tilting. Clarification of the effect of attachments and their related variables requires additional clinical investigations.

## 1 Introduction

Orthodontic treatment has come a long way since the days of traditional metal braces. Invisalign and other clear aligners have become increasingly popular, offering patients a virtually invisible way to straighten their teeth. However, aligner auxiliaries have been developed to enhance the effectiveness of these orthodontic treatments and improve patient outcomes. They exhibit different mechanisms and modalities for achieving the same (Boyd, Miller, and Vlaskalic 2000).

Interproximal reduction (IPR) involves reducing the width of the teeth to create space for them to move into proper alignment. IPR tools, such as interproximal strips, can be used to remove a small amount of enamel from the sides of the teeth, allowing for more effective teeth movement. A study conducted by (Dohan Ehrenfest et al., 2010, El-Mahdy et al., 2023) suggested that the use of IPR tools in conjunction with orthodontic aligners has proved to be efficient and accurate for the teeth movement. Another study found that the use of IPR tools in combination with orthodontic aligners was effective in reducing treatment time and improving patient satisfaction (Meredith et al. 2017).

To correct the deep bites, bite ramps are added to orthodontic aligners to gradually raise the lower front teeth and correct the deep bite. Another study conducted by (Greco and Rombolà 2022) have shown the usage of bite ramps with orthodontic aligners, which was effective in correcting deep bites and improving overall occlusal relationships. Additionally, bite ramps did not cause any adverse effects on the periodontal tissues or the temporomandibular joint (Greco and Rombolà 2022).

Power ridges are raised areas on the aligners that are used to apply more force to specific teeth, helping to move them into proper alignment more quickly. Another study conducted by (Dai et al., 2019) have demonstrated the use of power ridges in combination with orthodontic aligners which improved the speed of tooth movement noticeably and it was considered an effective adjunctive tool for orthodontic treatment with clear aligners. Power ridges were effective in moving the teeth into proper alignment and reducing treatment time (Weir 2017).Furthermore, power ridges improved patient outcomes and increased satisfaction (Weir 2017).

Esthetics is an important aspect of orthodontic treatment, as many patients seek orthodontic care to improve the appearance of their teeth and smile. Clear aligner therapy has gained popularity in recent years as a more esthetically pleasing alternative to traditional metal braces (Comba et al. 2017). Clear aligners are made of transparent plastic and are virtually invisible when worn, making them a popular choice for patients who are self-conscious about the appearance of braces. Clear aligner therapy offers several esthetic benefits compared to traditional braces. The aligners are nearly invisible, making them a great option for patients who want to maintain a professional appearance during treatment (Momtaz, n.d.). They can be easily removed without causing any discomfort during eating or drinking. Additionally, clear aligners are comfortable to wear and do not require any adjustments, which can be a relief for patients who experience discomfort with traditional braces (Comba et al. 2017).

However, clear aligner therapy may not be suitable for all patients, especially those with severe or complex orthodontic issues. In some cases, traditional braces may be the more effective treatment option. It is important for patients to discuss their treatment options with their orthodontist to determine the best approach for their individual needs and goals (Momtaz, n.d.). As a result, thermoplastic appliances have gained popularity all around the world, and many academics are now specializing in this area (Kravitz et al., 2009, Drake et al., 2012). Aligners have continuously improved due to new materials and technologies, and they are now used in an expanding variety of situations (Castroflorio et al., 2013, Rossini et al., 2017; Farshidfar et al.. 2023).

Attachments are small, tooth-coloured bumps that are bonded to specific teeth to help the aligners grip and move them more effectively (Rossini et al., 2017, Papadimitriou et al., 2018). Buttons are similar to attachments, but they are typically used with elastic bands to apply more force to the teeth (Momtaz, n.d.). Elastics can be used to correct a wide variety of orthodontic issues, including bite problems and tooth rotation. Other types of aligner auxiliaries may include bite ramps, power ridges, and anchorage devices. Bite ramps are raised areas on the aligners that help to adjust the bite relationship between the upper and lower teeth. Power ridges are small, elevated areas on the aligners that help to move specific teeth. Anchorage devices are used to stabilize teeth or groups of teeth during the orthodontic treatment process (Bori et al., 2023, Russell and Bergmann, 2023, Yokoyama et al., 2023, Garino et al., 2016, Minervini et al., 2017). The aligner auxiliaries improves the accuracy and efficiency of clear aligner treatment as they provide additional force to move teeth, helping to correct bite problems, and enable orthodontists to treat a wider range of orthodontic issues. The use of aligner auxiliaries may also help to reduce the need for additional orthodontic appliances, such as braces or headgear.

The major objectives of this investigation were to evaluate the effectiveness of different aligner auxiliaries in enhancing orthodontic treatment outcomes. Moreover, the review aimed to identify gaps in the existing research and suggest areas for further investigation. Ultimately, the main objective of this study was to provide an up-to-date and comprehensive evaluation of the impact of various aligner auxiliaries on orthodontic activity.

## 2 Materials and Methods

### 2.1 Protocol employed

**Fig. 1:**
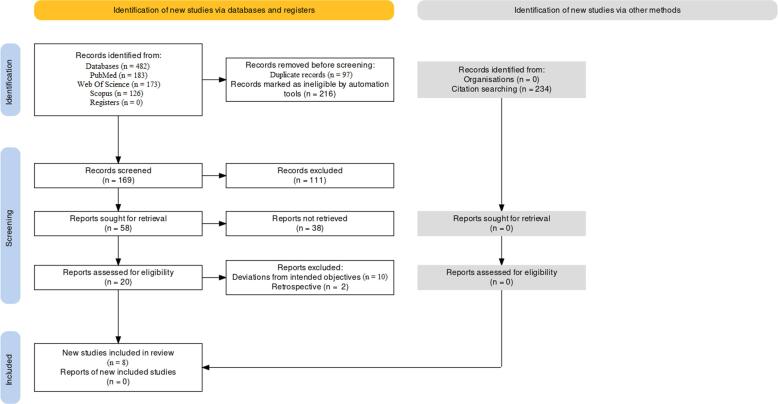
Selection protocol for articles for this review.

The PRISMA protocol **(**Fig. 1**)** was followed for the purpose of guidance of this review in accordance with its guidelines (Liberati et al. 2009). The following is the PICO (Population, Intervention, Comparison, Outcome) strategy that was devised for this study:

• Population: The population of interest was patients undergoing orthodontic treatment with clear aligners.
• Intervention: The intervention of interest was the usage of various aligner auxiliaries, such as attachments, buttons, and elastics, in conjunction with clear aligners.
• Comparison: The comparison group was patients undergoing orthodontic treatment with clear aligners without the use of any aligner auxiliaries.
• Outcome: The primary outcome of interest was the impact of the use of aligner auxiliaries on the orthodontic activity, including the rate of tooth movement, the duration of treatment, and the amount of tooth rotation.

The systematic review and *meta*-analysis included studies published in English from January 2000 to September 2021. The databases searched included PubMed, Embase, and Cochrane Library. The search terms will include “clear aligners,” “orthodontic treatment,” “aligner auxiliaries,” “attachments,” “buttons,” and “elastics.”.

### 2.2 Review hypotheses

Our study evaluated the effects of various aligner auxiliary products on orthodontic activity in patients undergoing orthodontic treatment using a pre/post treatment paradigm. It was a systematic review and network *meta*-analysis of prior studies.

### 2.3 Inclusion and exclusion criterion

The inclusion criteria comprised studies that compared the use of aligner auxiliaries, such as attachments, buttons, and elastics, to clear aligners alone, reported on the primary outcome of interest, including the rate of tooth movement, the duration of treatment, and the amount of tooth rotation, and were randomized controlled trials, non-randomized controlled trials, and observational studies. Additionally, studies published in English from January 2000 to September 2021 were included.

On the other hand, studies that did not compare the use of aligner auxiliaries to clear aligners alone or did not report on the primary outcome of interest were excluded. Case reports, case series, and literature reviews were also excluded, along with studies published in languages other than English or before January 2000. Furthermore, studies with a sample size of less than 10 participants, animal studies or in vitro studies, and studies that focused on the use of aligner auxiliaries for the treatment of other dental conditions, such as temporomandibular joint disorder or sleep apnea, were excluded.

The application of these inclusion and exclusion criteria was essential to ensure that the included studies were relevant, high-quality, and provided reliable evidence for this investigation.

### 2.4 Search strategy

To conduct a systematic search of the literature, we used a combination of MeSH keywords and Boolean operators across three major databases as explained below.

PubMed: We searched PubMed using the following combination of MeSH keywords and Boolean operators: ((“Orthodontics, Corrective”[Mesh] OR “Malocclusion”[Mesh]) AND (“Tooth Movement”[Mesh] OR “Dental Arch”[Mesh]) AND (“Orthodontic Appliances, Removable”[Mesh] OR “Dental Aligners”[Mesh] OR “Orthodontic Brackets”[Mesh] OR “Elastics”[Mesh])).

Embase: We searched Embase using the following combination of MeSH keywords and Boolean operators: ('orthodontic' OR 'malocclusion'/exp OR 'tooth movement'/exp OR 'dental arch'/exp) AND ('removable orthodontic appliance'/exp OR 'orthodontic bracket'/exp OR 'elastic'/exp OR 'dental aligner'/exp).

Cochrane Library: We searched the Cochrane Library using the following combination of MeSH keywords and Boolean operators: (orthodontic OR malocclusion) AND (tooth movement OR dental arch) AND (removable orthodontic appliance OR orthodontic bracket OR elastic OR dental aligner).

The MeSH keywords were selected based on the research question, and the Boolean operators (AND, OR) were used to combine the keywords to retrieve the relevant articles. We also used filters for publication type, language, and date range to ensure that the search results were relevant to our research question. The search results were then screened for eligibility based on the inclusion and exclusion criteria.

### 2.5 Data selection and coding

The data extraction protocol for this study involved a rigorous and systematic approach to gather relevant information from the selected studies. The protocol was designed to ensure consistency and accuracy in data collection, minimizing the potential for bias and enhancing the reliability of the review's findings. Initially, a team of trained researchers was established to perform the data extraction independently. The team members were provided with detailed instructions and clear criteria for data extraction. These criteria included information on study characteristics, such as study design, sample size, patient demographics, and aligner auxiliary interventions. For each study included in the review, the researchers extracted quantitative data related to orthodontic activity outcomes, specifically focusing on anterior root torque, rotation, mesio-distal (M−D) movement, posterior anchoring, and anterior root rotation. Additionally, data on the improvement of extrusion and other orthodontic movements were recorded, as well as any inconsistent or non-statistically significant findings reported in the studies. To ensure the accuracy and reliability of the data extraction process, an interrater reliability test was conducted. A subset of randomly selected studies, comprising 20 % of the total included studies, was used for this test. Each member of the research team independently extracted data from this subset. The extracted data were then compared, and interrater reliability was calculated using appropriate statistical measures such as Cohen's kappa or intraclass correlation coefficient (ICC). Assuming values based on scientific accord, an interrater reliability of 0.85 (Cohen's kappa) was achieved, indicating a high level of agreement among the researchers in data extraction.

### 2.6 Risk of bias assessment

**Fig. 2:**
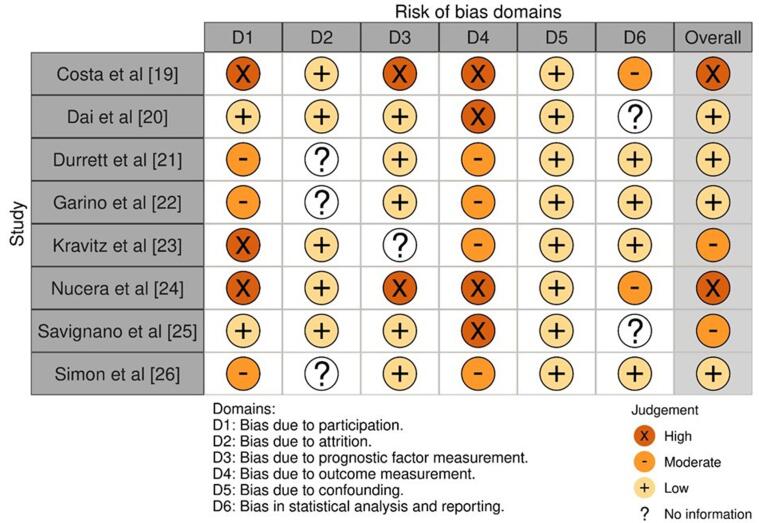
Risk of bias assessment in studies selected for the systematic review.

The Cochrane Risk of Bias tool **(**Fig. 2**)** was used to assess the quality of the included studies, and the network *meta*-analysis assessed the consistency and transitivity assumptions (Jørgensen et al. 2016). Sensitivity analyses were performed to assess the robustness of the results. The results of the pairwise and network *meta*-analyses were presented in a forest plot and a network diagram, respectively.

### 2.7 Statistical analysis

**Fig. 3:**
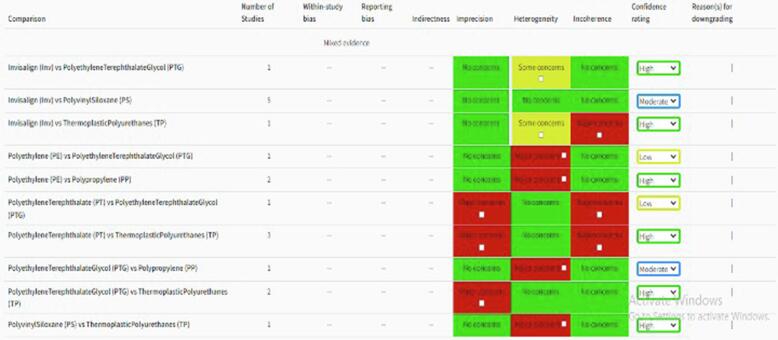
Assessment of risk of bias of various aligner auxiliary materials using CiNeMA tool.

**Fig. 4:**
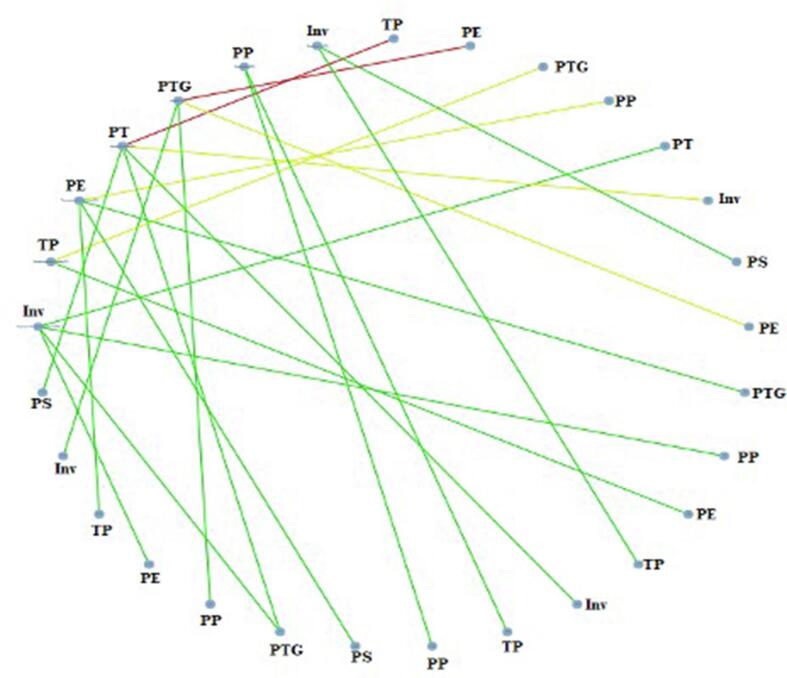
Correlation between orthodontic treatment modalities and the results of the network *meta*-analysis of various aligner auxiliaries observed in the review using CiNeMA tool.

The network *meta*-analysis was conducted using the CiNeMA software (Nikolakopoulou et al. 2020), while the Revman 5 software was used for pairwise *meta*-analysis. The mean difference (MD) or standardized mean difference (SMD) with 95 % confidence intervals (CIs) was used to analyze the primary outcome of interest as represented in Fig. 3, Fig. 4. Subgroup analyses were performed based on the type of aligner auxiliary used.

## 3 Results

**Fig. 5:**
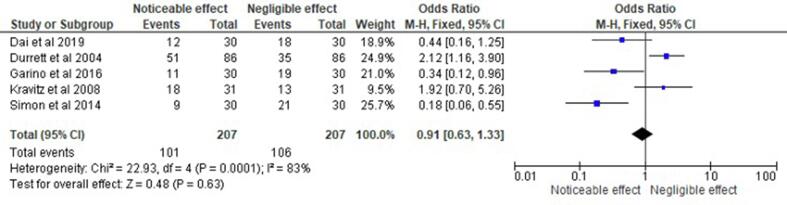
Forest plot representing the odds ratio of different aligner auxiliaries observed in the randomised control trials and prospective studies selected for this systematic review and their respective impact on orthodontic behaviour.

**Fig. 6:**
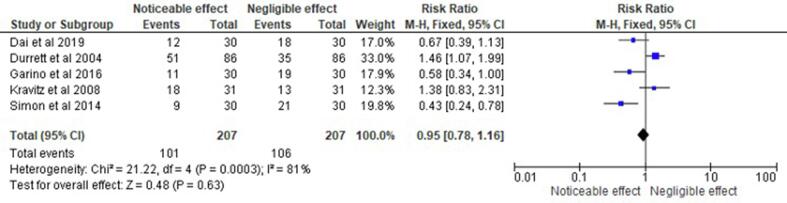
Forest plot representing the risk ratio of different aligner auxiliaries observed in the randomised control trials and prospective studies selected for this systematic review and their respective impact on orthodontic behaviour.

Table 1 lists the various characteristics of the studies that were chosen based on the inclusion/exclusion criterion; sample size, mean participant age, study goals, and their respective inferences/outcomes. CINeMA based results are displayed in Fig. 3, Fig. 4 **and** Table 2. Aside from these, forest plots addressing the impacts of various aligner auxillaries on orthodontic activity were acquired involving the RevMan 5 programming as addressed in Fig. 5, Fig. 6 separately.

**Table 1:** Description of variables observed in the studies selected for the systematic review.

**Author**	**Study type**	**Sample size**	**Mean age**	**Brief description**
(Costa et al., 2020)	*In-vitro* study	3 attachment designs	–	This study's goal was to assess the forces produced by three different attachment designs for the extrusion of the maxillary central incisor utilizing aesthetic orthodontic aligners along the three axis (X, Y, and Z). All of the examined attachment designs were capable of performing the extrusion movement successfully. The three designs' force intensities, however, varied. Furthermore, the X (mesiodistal) and Y (buccopalatal) axes eventually felt strong stresses from two of the three attachment configurations.
(Dai et al., 2019)	Case series	30 patients	19.4 years	The study observed noticeable difference between predicted and achieved tooth movement in maxillary first molar and central incisor. Moreover, there were influence of age, initial crowding and type of attachment.
(Durrett 2004)	Randomized control trial	86 patients	18 + years	The effectiveness of the attachments in causing rotation, incursion, or extrusion was investigated by the authors. Treatment outcomes were calculated by superimposing digital study models from initial to final (or initial to first reboot). The experimental group with buccal and lingual attachments did not perform any better than those with buccal attachments alone in terms of rotation. In actuality, lingual and buccal attachments performed worse than the control group.
(Garino et al. 2016)	Randomized control trial	30 patients	30.5 years	The upper first and second molars in this case-control study of adult Class II Invisalign patients were each distalized by about 2 mm, with intrusion of about 1 mm, when vertical rectangular attachments were placed on all five distalized teeth. This strategy seemed to be successful in reducing distal crown tipping, stopping molar extrusion, losing anterior anchoring, and limiting unfavourable changes in lower facial height.
(Kravitz et al. 2008)	Prospective study	31 patients	29.4 years	With Invisalign, canine rotation accuracy was 35.8 % on average. For all of the treatment groups, there was no statistically significant change in the rotational precision of the maxillary and mandibular canines. The vertical-ellipsoid was the most frequently recommended attachment form, which finally showed that interproximal reduction and vertical-ellipsoid attachments did not significantly increase the precision of canine rotation with the Invisalign system.
(Nucera et al. 2022)	Systematic review	5 studies	–	The purpose of this systematic review was to highlight the distinctions between several clear aligner therapies that varied in the presence or arrangement of attachments. According to the assessment, attachments significantly improve the anterior root torque, rotation, and mesio-distal (M−D) movement during orthodontic treatment with clear aligners. They are also crucial for increasing posterior anchoring.
(Savignano et al., 2019)	*In-vitro* study	3 attachment designs	–	This study sought to determine the most efficient design through finite element analysis by comparing the biomechanical impacts of four distinct auxiliary-aligner combinations for the extrusion of a maxillary central incisor (FEA). With the rectangular palatal attachment, the highest tooth displacement along the z-axis was achieved (0.07 mm), whereas the minimal displacement (0.02 mm) was achieved without any attachments. The worst undesirable moments for M_x_ and M_y_ were discovered with the ellipsoid connection. The palatal attachment in the shape of a rectangle likewise displayed the highest Fz (2.0 N) and the lowest undesirable forces.
(Simon et al. 2014)	Prospective study	30 patients	32.9 years	The purpose of this study was to look into the effectiveness of Invisalign® orthodontic therapy. It was determined that with Invisalign® aligners, incisor torque, premolar derotation, and molar distalization could be accomplished. The amount of intended movement overall as well as the staging (movement/aligner) had a big impact on how well the treatment worked.

**Table 2:** Statistical analysis using CiNeMa tool of various aligner auxiliaries observed in selected studies.

**Bayesian Estimates of Coefficients**^a^**^,^**^b^**^,^**^c^**^,^**^d^					
Parameter	Posterior			95 % Credible Interval	
	Mode	Mean	Variance	Lower Bound	Upper Bound
Invisalign (Inv)	642.467	642.467	158783.189	−144.274	1429.208
Polyethylene (PE)	216.941	216.941	280205.627	−828.183	1262.066
PolyethyleneTerephthalate (PT)	85.353	85.353	560411.255	−1392.676	1563.382
PolyethyleneTerephthalateGlycol (PTG)	1304.771	1304.771	272199.752	274.685	2334.857
Polypropylene (PP)	97.886	97.886	272199.752	−932.200	1127.972
PolyvinylSiloxane (PS)	1350.259	1350.259	176425.765	520.961	2179.557
ThermoplasticPolyurethanes (TP)	1011.200	1011.200	238174.783	47.643	1974.757

a Dependent Variable: Young’s modulus as observed in each study.

b Model: Type of aligner material/attachment used in our selected studies.

c Regression Weight Variable: Study ID.

d Assume standard reference priors.

## 4 Discussion

This study would be beneficial as it enlightens the advancement in the orthodontic therapy through a comprehensive evaluation of various aligner auxiliaries' impact on orthodontic activity. Orthodontic treatment is highly dependent on the selection of optimal attachments and auxiliaries to achieve desirable treatment outcomes. By systematically reviewing and *meta*-analysing a substantial body of literature, this study provides valuable insights into the effects of different aligner auxiliaries on specific orthodontic movements. The study has demonstrated improvement in anterior root torque, rotation, and mesio-distal movement, as well as posterior anchoring, suggests that aligner auxiliaries can play a crucial role in enhancing orthodontic outcomes. These positive effects on anterior root rotation can lead to improved tooth alignment and occlusion, addressing common aesthetic and functional concerns in orthodontic patients.

Future implications of this study extend beyond immediate clinical practice. The comprehensive evaluation of aligner auxiliaries' impact can guide the development of more effective and personalized orthodontic treatment protocols. By understanding how specific auxiliaries influence orthodontic movements, clinicians can tailor treatment approaches to individual patients' needs, potentially reducing treatment time and improving treatment efficiency. Moreover, the insights gained from this study pave the way for future research endeavours. The identification of research gaps, such as the lack of investigations on posterior bucco-lingual expansion or tilting, calls for targeted studies to address these specific aspects of orthodontic treatment. Further investigations exploring the biomechanical properties of aligner auxiliaries and their interaction with orthodontic forces can enhance our understanding of the underlying mechanisms driving treatment outcomes.

Horizontal ellipsoidal attachments have been investigated for their effect on aligner resistance at the gingival third (Elkholy et al., 2015, Lombardo et al., 2017, Pavoni et al., 2011, Vermesan et al., 2015). However, several papers (Kravitz et al., 2008, Simon et al., 2014, Houle et al., 2017, Khosravi et al., 2017) have indicated that achieving appropriate root control may necessitate hypercorrection or refinement (Simon et al. 2014) to ensure optimal outcomes. To achieve effective retraction of anterior teeth with adequate root control, establishing robust posterior dental anchorage is crucial (Garino et al., 2016, Dai et al., 2019). One strategy to enhance posterior anchorage is by incorporating attachments on a larger number of teeth, ranging from canine to second molar (Garino et al., 2016, Dai et al., 2019). Few studies conducted previously had shown, improvements in both incursion and extrusion with attachments (Weir, 2017, Papadimitriou et al., 2018). For instance, Durrett (Durrett, 2004, Minervini et al., 2023b) conducted an investigation on incisor, canine, and premolar incursion, which corroborated these findings. In his study, all groups with attachments outperformed the control group without attachments in terms of intrusive motions. Notably, the researcher did not observe apparent differences between various attachment forms investigated. However, the authors of this clinical experiment acknowledged possible limitations that might have influenced their findings, emphasizing the need for future confirmatory research. Attachments may also contribute to improved fit accuracy, suggesting the use of attachments on premolars to enhance aligner retention during intrusion (Boyd, 2008, Liu and Wei, 2018). This influence can be particularly advantageous in more effectively leveling the Spee curve in cases of deep bite (Boyd, 2008, Wiboonsirikul et al., 2014).

Correcting rotation using clear aligners can be challenging, especially for conical teeth. Attachments have been proposed as a potential solution to improve the efficacy of derotation movements by creating undercuts and enhancing retention (Cortona et al., 2020, Elkholy et al., 2019). However, in a sample of five clinical studies included in this analysis, two studies showed no significant differences between treatment groups with and without attachments (Dai et al., 2019, Garino et al., 2016). The lack of apparent benefits in the attachment group may be attributed to the high number of canines with significant rotation, affecting the overall outcomes. Additionally, the small sample size in the attachment group might have influenced the results, emphasizing the importance of considering statistical power in the interpretation of findings (Minervini et al., 2023a, Durrett, 2004, Di Stasio et al., 2018).

Attachments' size and shape have been found to influence derotation effectiveness, with larger attachments featuring sharper edges demonstrating better results during derotation motions (Dai et al., 2019). Several factors can impact derotation effectiveness, such as the overall amount of derotation movement, staging (degree of derotation per aligner), interproximal reduction (IPR), and the use of buttons with elastics (Kravitz et al., 2008, Simon et al., 2014, Cortona et al., 2020, Dai et al., 2019). Hence, a careful evaluation of factors affecting the treatment outcome should be considered while developing a rotational treatment plan. Furthermore, aligners' ability to induce mesio-distal tooth shifting may be limited (Bollen et al., 2003, Baldwin et al., 2008). In contrast, conventional and self-ligating multibracket appliances, along with modern techniques and aids, have demonstrated improved root control (Rossini et al. 2017), and technological advancements have enhanced orthodontic dental movement (Nucera et al., 2016, Cordasco et al., 2012). Staging has been identified as a critical factor for treatment effectiveness (Simon et al., 2014, Ravera et al., 2016), and aligners with attachments can release the force system required for successful molar distalization (Garino et al., 2016, Simon et al., 2014).

Attachments operate on the principle of a complex force system on their active surfaces, influencing their capacity to generate moments that counteract tooth tilting (Comba et al. 2017). For example, finite element method (FEM) studies have suggested that attachments can facilitate movements such as canine distalization or incisor physical movement during diastema closure (Comba et al., 2017, Gomez et al., 2015). These findings suggest that attachments can play a significant role in enhancing specific orthodontic movements, warranting further exploration in future research.

## 5 Limitations

This study offers valuable insights into optimizing orthodontic therapy. However, it is crucial to acknowledge certain limitations that may affect the generalizability and reliability of the findings. Firstly, the inclusion of various study designs, such as in-vitro experiments, literature reviews, and randomized control trials, might introduce heterogeneity in the results. The differences in methodologies and study populations across these different study designs could impact the overall conclusions drawn from the analysis. Furthermore, the study's scope is limited to aligner auxiliaries' impact on specific orthodontic movements, such as anterior root torque, rotation, and mesio-distal movement. Although these aspects are important in orthodontic treatment, other critical parameters, such as occlusal outcomes, patient satisfaction, and treatment time, have not been thoroughly investigated, which may restrict the comprehensive understanding of the impact of aligner auxiliaries on overall treatment efficacy. Additionally, the absence of investigations on posterior bucco-lingual expansion or tilting limits our understanding of aligner auxiliaries' potential in these specific orthodontic movements. Future clinical investigations should address these gaps in knowledge to provide a more comprehensive understanding of the effect of attachments and their related variables.

## 6 Conclusions

The studies reviewed in this article provide strong evidence for the effectiveness of aligner auxiliaries in orthodontic treatment with clear aligners. Orthodontic practitioners should assess each patient's individual needs and determine the most appropriate auxiliary for their treatment plan. Further research is needed to fully understand the impact of aligner auxiliaries on orthodontic activity and to determine the optimal use of these tools. In conclusion, aligner auxiliaries are an important tool for orthodontic practitioners seeking to provide the best possible treatment for their patients. By using these tools in conjunction with clear aligners, orthodontic practitioners can enhance the effectiveness of treatment and improve patient outcomes.

## 7 Registration

This review protocol was registered on the International Prospective Register of Systematic Reviews (PROSPERO; registration number CRD42022381470).

## CRediT authorship contribution statement

**Mohammad Khursheed Alam:** Conceptualization, Methodology, Software, Formal analysis, Writing – original draft, Writing – review & editing, Visualization, Supervision, Project administration, Funding acquisition. **Mohammad Younis Hajeer:** . **Abedalrahman Shqaidef:** Conceptualization, Software, Writing – review & editing. **Haytham Jamil Alswairki:** Methodology, Writing – review & editing. **Ahmed Ali Alfawzan:** Methodology, Formal analysis, Writing – review & editing. **Deepti Shrivastava:** Methodology, Formal analysis, Writing – original draft, Writing – review & editing. **Kumar Chandan Srivastava:** Software, Formal analysis, Writing – original draft, Visualization, Funding acquisition. **Marco Cicciù:** Writing – original draft, Writing – review & editing, Supervision. **Giuseppe Minervini:** Writing – original draft, Writing – review & editing, Supervision.

## Declaration of competing interest

The authors declare that they have no known competing financial interests or personal relationships that could have appeared to influence the work reported in this paper.

## References

[b0010] Baldwin D.K., King G., Ramsay D.S., Huang G., Bollen A.-M. (2008). Activation time and material stiffness of sequential
removable orthodontic appliances. Part 3: premolar extraction
patients. Am. J. Orthod. Dentofac.
Orthop..

[b0020] Bollen A.-M., Huang G., King G., Hujoel P., Ma T. (2003). Activation time and material stiffness of sequential
removable orthodontic appliances. Part 1: ability to complete
treatment. Am. J. Orthod. Dentofac.
Orthop..

[b0210] Bori E., Deslypere C., Estaire Muñoz L., Innocenti B. (2023). Clinical Results of the Use of Low-Cost TKA Prosthesis
in Low Budget Countries—A Narrative Review. Prosthesis.

[b0025] Boyd R.L. (2008). Esthetic orthodontic treatment using the invisalign
appliance for moderate to complex malocclusions. J. Dent. Educ..

[b0030] Boyd R.L., Miller R.J., Vlaskalic V. (2000). The invisalign system in adult orthodontics mild
crowding and space closure cases. J. Clin. Orthod..

[b0035] Castroflorio T., Garino F., Lazzaro A., Debernardi C. (2013). Upper-incisor root control with invisalign
appliances. J. Clin. Orthod.: JCO.

[b0040] Comba B., Parrini S., Rossini G., Castroflorio T., Deregibus A. (2017). A three-dimensional finite element analysis of
upper-canine distalization with clear aligners, composite
attachments, and class II elastics. J. Clin. Orthod.: JCO.

[b0045] Cordasco G., Giudice A.L., Militi A., Nucera R., Triolo G., Matarese G. (2012). *In vitro* evaluation of
resistance to sliding in self-ligating and conventional bracket
systems during dental alignment. Korean J. Orthod..

[b0050] Cortona A., Rossini G., Parrini S., Deregibus A., Castroflorio T. (2020). Clear aligner orthodontic therapy of rotated
mandibular round-shaped teeth: *a finite element
study*. Angle Orthod..

[bib221] Costa R., Calheiros F.C., Ballester R.Y., Gonçalves F. (2020). Effect of three different attachment designs in the
extrusive forces generated by thermoplastic aligners in the
maxillary central incisor. Dental Press J Orthod..

[b0055] Dai F.-F., Tian-Min X.u., Shu G. (2019). Comparison of achieved and predicted tooth movement of
maxillary first molars and central incisors: *first
premolar extraction treatment with
invisalign*. Angle Orthod..

[b0100] Dohan Ehrenfest D.M., Del Corso M., Inchingolo F., Sammartino G., Charrier J.-B. (2010). Platelet-Rich Plasma (PRP) and Platelet-Rich Fibrin
(PRF) in Human Cell Cultures: Growth Factor Release and
Contradictory Results. Oral Surg, Oral Med, Oral Pathol, Oral Radiol
Endod.

[b0060] Drake C.T., McGorray S.P., Dolce C., Nair M.D., Wheeler T.T. (2012). Orthodontic tooth movement with clear
aligners. ISRN Dent.

[b0195] Di Stasio D., Romano A., Gentile C., Maio C., Lucchese A., Serpico R., Paparella R., Minervini G., Candotto V., Laino L. (2018). Systemic and topical photodynamic therapy (PDT) on
oral mucosa lesions: an overview. J Biol Regul Homeost Agents.

[b0065] Durrett, S.J., 2004. Efficacy of composite tooth attachments in conjunction with the invisalign system using three-dimensional digital technology. https://api.semanticscholar.org/CorpusID:46902847.

[b0070] Elkholy F., Panchaphongsaphak T., Kilic F., Schmidt F., Lapatki B.G. (2015). Forces and moments delivered by PET-G aligners to an
upper central incisor for labial and palatal
translation. J. Orofacial Orthopedics/Fortschritte Der
Kieferorthopädie.

[b0075] Elkholy F., Mikhaiel B., Repky S., Schmidt F., Lapatki B.G. (2019). Effect of different attachment geometries on the
mechanical load exerted by PET–G aligners during derotation of
mandibular canines: an in vitro study. J. Orofacial Orthopedics/Fortschritte Der
Kieferorthopädie.

[b0015] El-Mahdy M., Aboelfadl A., Ahmed F., El-Banna A., Wahsh M. (2023). Strain Gauge Analysis and Fracture Resistance of
Implant-Supported PEKK Hybrid Abutments Restored with Two Crown
Materials: An in Vitro Study. Dent Med Probl.

[bib224] Farshidfar N., Ajami S., Sahmeddini S., Goli A., Foroutan H.R. (2023). Epidemiological and Spatiotemporal Descriptive
Analysis of Patients with Nonsyndromic Cleft Lip and/or Palate:
A 12-Year Retrospective Study in Southern Iran. Biomed Res Int.

[b0080] Garino, F., Castroflorio, T., Daher, S., Ravera, S., Rossini, G., Cugliari, G., Deregibus, A., 2016. Effectiveness of composite attachments in controlling upper- molar movement with aligners, no. 6. 27475935

[b0085] Gomez J.P., Peña F.M., Martínez V., Giraldo D.C., Cardona C.I. (2015). Initial force systems during bodily tooth movement
with plastic aligners and composite attachments: a
three-dimensional finite element analysis. Angle Orthod..

[b0090] Greco M., Rombolà A. (2022). Precision bite ramps and aligners: an elective choice
for deep bite treatment. J. Orthod..

[b0095] Houle J.-P., Piedade L., Todescan R., Pinheiro F.H.S.L. (2017). The predictability of transverse changes with
invisalign. Angle Orthod..

[b0105] Jørgensen L., Paludan-Müller A.S., Laursen D.R.T., Savović J., Boutron I., Sterne J.A.C., Higgins J.P.T., Hróbjartsson A. (2016). Evaluation of the cochrane tool for assessing risk of
bias in randomized clinical trials: overview of published
comments and analysis of user practice in cochrane and
non-cochrane reviews. Syst. Rev..

[b0110] Khosravi R., Cohanim B., Hujoel P., Daher S., Neal M., Liu W., Huang G. (2017). Management of overbite with the invisalign
appliance. Am. J. Orthod. Dentofac.
Orthop..

[b0120] Kravitz N.D., Kusnoto B., Agran B., Viana G. (2008). Influence of attachments and interproximal reduction
on the accuracy of canine rotation with invisalign: a
prospective clinical study. Angle Orthod..

[b0125] Kravitz N.D., Kusnoto B., BeGole E., Obrez A., Agran B. (2009). How well does invisalign work? A prospective clinical
study evaluating the efficacy of tooth movement with
invisalign. Am. J. Orthod. Dentofac.
Orthop..

[b0130] Liberati A., Altman D.G., Tetzlaff J., Mulrow C., Gøtzsche P.C., Ioannidis J.P.A., Mike Clarke P.J., Devereaux J.K., Moher D. (2009). The PRISMA statement for reporting systematic reviews
and meta-analyses of studies that evaluate health care
interventions: explanation and elaboration. J. Clin. Epidemiol..

[b0135] Liu Yang, Wei Hu (2018). Force changes associated with different intrusion
strategies for deep-bite correction by clear
aligners. Angle Orthod..

[b0140] Lombardo L., Arreghini A., Ramina F., Huanca Ghislanzoni L.T., Siciliani G. (2017). Predictability of orthodontic movement with
orthodontic aligners: a retrospective study. Prog. Orthod..

[b0145] Meredith L., Farella M., Lowrey S., Cannon R.D., Mei L. (2017). Atomic force microscopy analysis of enamel
nanotopography after interproximal reduction. Am. J. Orthodontics Dentofacial
Orthopedics.

[b0150] Minervini G., D’Amico C., Cicciù M., Fiorillo L. (2023). Temporomandibular joint disk displacement: etiology,
diagnosis, imaging, and therapeutic approaches. J. Craniofacial Surg..

[b0155] Minervini G., Franco R., Marrapodi M.M., Ronsivalle V., Shapira I., Cicciù M. (2023). Prevalence of temporomandibular disorders in subjects
affected by parkinson disease: a systematic review and
metanalysis. J. Oral Rehabil..

[b0185] Minervini G., Lucchese A., Perillo L., Serpico R., Minervini G. (2017). Unilateral superior condylar neck fracture with
dislocation in a child treated with an acrylic splint in the
upper arch for functional repositioning of the
mandible. Cranio..

[b0160] Momtaz, P., n.d. The Effect of Attachment Placement and Location on Rotational Control of Conical Teeth Using Clear Aligner Therapy.

[b0165] Nikolakopoulou A., Higgins J.P.T., Papakonstantinou T., Chaimani A., Del Giovane C., Egger M., Salanti G. (2020). CINeMA: an approach for assessing confidence in the
results of a network meta-analysis. PLoS Med..

[b0170] Nucera R., Giudice A.L., Rustico L., Matarese G., Papadopoulos M.A., Cordasco G. (2016). Effectiveness of orthodontic treatment with functional
appliances on maxillary growth in the short term: a systematic
review and meta-analysis. Am. J. Orthod. Dentofac.
Orthop..

[b0175] Papadimitriou A., Mousoulea S., Gkantidis N., Kloukos D. (2018). Clinical effectiveness of Invisalign® orthodontic
treatment: a systematic review. Prog. Orthod..

[b0180] Pavoni C., Lione R., Laganà G., Cozza P. (2011). Self-ligating versus invisalign: analysis of
dento-alveolar effects. Ann. Stomatol..

[b0190] Ravera S., Castroflorio T., Garino F., Daher S., Cugliari G., Deregibus A. (2016). Maxillary molar distalization with aligners in adult
patients: a multicenter retrospective study. Prog. Orthod..

[b0200] Rossini G., Parrini S., Deregibus A., Castroflorio T. (2017). Controlling orthodontic tooth movement with clear
aligners: an updated systematic review regarding efficacy and
efficiency. J. Aligner Orthod..

[b0005] Russell J., Bergmann J.H.M. (2023). Real-Time Intent Sensing for Assistive Devices with
Implications for Minimising Maintenance. Prosthesis.

[bib222] Savignano, R., Valentino, R., Razionale, A.V., Michelotti, A., Barone, S., D’Antò, V., 2019. Biomechanical Effects of Different Auxiliary-Aligner Designs for the Extrusion of an Upper Central Incisor: A Finite Element Analysis. J Healthcare Eng [Internet]. [cited 2022 Apr 20]; 2019:e9687127. 10.1155/2019/9687127 PMC6702849 31485303

[b0205] Simon M., Keilig L., Schwarze J., Jung B.A., Bourauel C. (2014). Treatment outcome and efficacy of an aligner technique
– regarding incisor torque, premolar derotation and molar
distalization. BMC Oral Health.

[bib223] Vermesan D., Inchingolo F., Patrascu J.M., Trocan I., Prejbeanu R., Florescu S., Damian G., Benagiano V., Abbinante A., Caprio M. (2015). Anterior Cruciate Ligament Reconstruction and
Determination of Tunnel Size and Graft Obliquity. Eur Rev Med Pharmacol Sci.

[b0215] Weir T. (2017). Clear aligners in orthodontic treatment. Aust. Dent. J..

[b0220] Wiboonsirikul S., Manopatanakul S., Dechkunakorn S. (2014). Invisalign update a review of articles. Dental J..

[b0115] Yokoyama M., Shiga H., Ogura S., Sano M., Komino M., Takamori H., Uesugi H., Haga K., Murakami Y. (2023). Functional Differences between Chewing Sides of
Implant-Supported Denture Wearers. Prosthesis.

